# Outgroup effects on root position and tree topology in the AFLP phylogeny of a rapidly radiating lineage of cichlid fish^☆^

**DOI:** 10.1016/j.ympev.2013.09.005

**Published:** 2014-01

**Authors:** Paul C. Kirchberger, Kristina M. Sefc, Christian Sturmbauer, Stephan Koblmüller

**Affiliations:** Department of Zoology, Karl-Franzens-University Graz, Universitätsplatz 2, A-8010 Graz, Austria

**Keywords:** Ancient incomplete lineage sorting, Cichlidae, Limnochromini, Outgroup rooting, Rapid radiation, Lake Tanganyika

## Abstract

Phylogenetic analyses of rapid radiations are particularly challenging as short basal branches and incomplete lineage sorting complicate phylogenetic inference. Multilocus data of presence-absence polymorphisms such as obtained by AFLP genotyping overcome some of the difficulties, but also present their own intricacies. Here we analyze >1000 AFLP markers to address the evolutionary history of the Limnochromini, a cichlid fish lineage endemic to Lake Tanganyika, and to test for potential effects of outgroup composition on tree topology. The data support previous mitochondrial evidence on the tribe’s taxonomy by confirming the polyphyly of the genus *Limnochromis* and – in contradiction to a recent taxonomic revision – nesting the genus *Greenwoodochromis* within the Limnochromini. Species relationships suggest that ecological segregation occurred during the rapid basal radiation of the Limnochromini. The large phylogenetic distance between candidate outgroup taxa and the Limnochromini radiation caused random outgroup effects. Bootstrap support for ingroup nodes was lower in outgroup-rooted than in midpoint-rooted trees, and root positions and ingroup tree topologies varied in response to the composition of the outgroup. These observations suggest that the predisposition for homoplastic evolution makes AFLP-based phylogenetic analyses particularly susceptible to random biases introduced by too-distant outgroup taxa.

## Highlights

• AFLPs resolve the phylogeny of Lake Tanganyika’s benthic deepwater cichlid lineage.
• The recently proposed tribe Greenwoodochromini is nested within the Limnochromini.
• The Limnochromini underwent rapid initial radiation into eco-morphologically distinct lineages.
• Large phylogenetic distances between outgroup and ingroup taxa cause random outgroup effects.

## 1 Introduction

Adaptive radiations such as the Darwin’s finches on the Galapagos archipelago, the Hawaiian silver swords, the Caribbean anoles lizards, and the cichlid fish of the East African Great Lakes provide opportunities for studying the processes underlying rapid speciation (Schluter, 2000; Brakefield, 2006; Salzburger, 2009). Yet, resolving the phylogeny of adaptive radiations still remains a challenge. Due to the extreme rapidity of the radiations, lineage sorting often lags behind cladogenesis, complicating phylogenetic inference based on single or a few molecular markers (Pamilo and Nei, 1988; Maddison and Knowles, 2006). Furthermore, the short basal branches typical for rapid radiations can make the phylogenetic reconstructions sensitive to the choice of outgroup taxa. In particular if the phylogenetic distance between outgroup and ingroup taxa is large, there is a high likelihood of homoplasy between ingroup and outgroup taxa. Consequently, the outgroup may attach randomly to the ingroup and bias the inferred ingroup tree topology (Wheeler, 1990; Huelsenbeck et al., 2002; Rota-Stabelli and Telford, 2008; Rosenfeld et al., 2012). The outgroup effect on tree topology is well established for sequence data, but has so far received no attention in connection with multilocus binary presence–absence data such as those obtained by AFLP genotyping. However, the elevated potential for homoplasy in this type of data (Althoff et al., 2007; García-Pereira et al., 2010) implies that random outgroup effects on AFLP tree topologies are at least as likely as in analyses of DNA sequences. Despite certain other AFLP-specific shortcomings, which are associated with low information content, dominance and phenetics (e.g. García-Pereira et al., 2010), AFLP data nonetheless harbor potential for improved species tree approximation as the phylogenetic signal is integrated from numerous loci distributed throughout the genome, thus reducing the confounding effects of incomplete lineage sorting and hybridization on phylogenetic inference (Koopman, 2005). Consequently, AFLP markers are frequently being applied to taxa originating from rapid radiations (Ogden and Thorpe, 2002; Schliewen and Klee, 2004; Herder et al., 2006; Fink et al., 2010; Geiger et al., 2010; Koblmüller et al., 2010; Sturmbauer et al., 2010; Joyce et al., 2011).

The present study addresses the phylogeny of the Limnochromini (Cichlidae, Perciformes), a tribe of cichlid fish endemic to Lake Tanganyika. The deep-water habitat of this East African rift lake formed 5–6 million years ago (Tiercelin and Mondeguer, 1991) and provided a prolific environment for intralacustrine speciation (Koblmüller et al., 2008a). The Limnochromini originated in the course of a rapid radiation into several mouthbrooding cichlid tribes (the C-lineage, Clabaut et al., 2005), and subsequently diversified into 10 benthic, mainly deepwater-dwelling (up to depths >100 m), species (Coulter, 1991; Konings, 1998; Duftner et al., 2005). Previous work on the Limnochromini entailed repeated taxonomic revisions. Initially, the tribe included 13 species in 8 genera, *Benthochromis*, *Baileychromis*, *Gnathochromis*, *Greenwoodochromis*, *Limnochromis*, *Reganochromis*, *Tangachromis* and *Triglachromis* (Poll, 1986). Extending the morphological data, Takahashi (2003) erected two new tribes, the Benthochromini and the Greenwoodochromini, for the corresponding genera, and excluded *Gnathochromis pfefferi* from the Limnochromini. The establishment of the Benthochromini and the exclusion of *G. pfefferi* were later corroborated by mtDNA data, whereas the validity of Greenwoodochromini as a separate tribe was rejected (Duftner et al., 2005; Fig. 1A). Since questions on the taxonomy and evolutionary history of the Limnochromini have not been fully settled by the existing data, additional information was sought by employing AFLP markers in the present study. The phylogenetically unresolved radiation of the C-lineage, from which the Limnochromini originate (Clabaut et al., 2005), made it difficult to choose a suitable set of outgroup taxa from among the C-lineage tribes. Because of the large phylogenetic distance between the Limnochromini and the candidate outgroup taxa (Duftner et al., 2005), particular attention was paid to the effect of outgroup composition on ingroup topology.

## 2 Materials and methods

### 2.1 Ingroup and outgroup taxa

The Limnochromini were represented by 31 specimens representing all described species of the tribe, except for the small deepwater-dwelling *Tangachromis dhanisi*, which is seldom caught and rarely found in the aquarium trade. Outgroup effects were examined by comparing a midpoint-rooted tree with trees rooted with different sets of outgroup taxa, which were assembled from a pool of 17 species in six tribes representing the entire C-lineage (Takahashi and Koblmüller, 2011; Muschick et al., 2012; see “Phylogenetic analyses”).

Fish were either caught on site using gill nets, acquired from aquarium trade or bought from local fishermen (Supplementary table 1).

### 2.2 DNA extraction and AFLP data collection

DNA extraction and AFLP genotyping (ten primer combinations for selective amplification: *EcoR*I-ACA/*Mse*I-CAA, *EcoR*I-ACA/*Mse*I-CAG, *EcoR*I-ACA/*Mse*I-CAC, *EcoR*I-ACA/*Mse*I-CAT, *EcoR*I-ACT/*Mse*I-CAA, *EcoR*I-ACT/*Mse*I-CAG, *EcoR*I-ACT/*Mse*I-CAC, *EcoR*I-ACT/*Mse*I-CAT, *EcoR*I-ACC/*Mse*I-CAA, *EcoR*I-ACT/*Mse*I-CAC) were performed as described in Egger et al. (2007). Initial scoring of electropherograms (size range of 50–300 bps for each primer combination; bin width was adjusted manually for each peak) was done using GeneMapper 3.7 (Applied Biosystems). AFLPScore 1.4a (Whitlock et al., 2008) was then used to convert the un-normalized peak-height data into a presence/absence matrix. 20 replicate samples were used to estimate the mismatch error rate, and a rate of <2% was set as threshold for the inclusion of data in the final matrix.

### 2.3 Phylogenetic analyses

PAUP 4.05b (Swofford, 2000) was used to construct a neighbor joining (NJ) tree based on Nei-Li distances (Nei and Li, 1979) and estimate statistical support from 1000 bootstrap replicates. To test for effects of outgroup composition, analyses were conducted with only ingroup taxa (the “ingroup dataset”) and with eight different sets of outgroup taxa, i.e. the “full dataset” using the complete outgroup (17 taxa from six tribes, encompassing the diversity of the entire C-lineage), and seven datasets with smaller outgroups (Ectodini; a monophyletic clade of Perissodini + Benthochromini + Cyprichromini; Cyprichromini; Benthochromini; Perissodini; Cyphotilapiini; Tropheini). For the ingroup dataset, a Neighbor-Net analysis based on a Nei and Li (1979) distance matrix was conducted in SplitsTree v.4.10 (Huson and Bryant, 2006) as an explorative approach for identifying conflicting phylogenetic signal in the data.

To test for consistency between mtDNA- and AFLP-based tree topologies, we evaluated the fit of the mtDNA sequence data (Duftner et al., 2005) to the unrooted NJ tree topology derived from ingroup AFLP data, by constraining a maximum likelihood (ML) tree search with the substitution model GTR + I + G (Duftner et al., 2005) to the AFLP tree. The Shimodaira-Hasegawa (SH) test (Shimodaira and Hasegawa, 1999; full optimization, 10,000 bootstrap replicates; implemented in PAUP) was then used to test whether the likelihood of the mtDNA data was significantly lower under the AFLP-NJ topology than under an unconstrained topology optimized from the mtDNA data.

## 3 Results

### 3.1 Phylogenetic reconstruction of the ingroup dataset

Phylogenetic analyses were based on 1128 AFLP marker loci. Bootstrap support for the monophyly of all species and for the reconstructed species clades was relatively good and ranged from 71% to 100% (Fig. 1B). Polyphyly of the genus *Limnochromis* was confirmed, as *L. auritus* was most closely related to *T. otostigma*, and *L. abeelei* and *L. staneri* clustered with the genus *Greenwoodochromis*. *Gnathochromis permaxillaris* resulted as sister group to the *L. abeelei*/*L. staneri*/*Greenwoodochromis* clade and *B. centropomoides* plus *R. calliurus* constituted a well supported monophylum. Mid-point rooting placed *T. otostigma *+ *L. auritus* as sister to the remaining limochromines. The SH-test (*p* = 0.059) revealed no significant differences between the AFLP topology of the ingroup dataset and the phylogenetic tree reconstructed from mitochondrial DNA sequence data by Duftner et al. (2005).

The phylogenetic relationships inferred by the NeighborNet analysis (Fig. 1C; least squares fit value = 95.633) were consistent with the tree-based inferences. Most of the internal splits in the network are represented by boxes, which is indicative of conflicting signals in the data. Incomplete or differential lineage sorting of marker loci as well as reticulation due to hybridization can underlie these ambiguities.

### 3.2 Outgroup effects

The NJ analysis of the full dataset (Fig. 2) confirmed the monophyly of the tribe Limnochromini (including the Greenwoodochromini). In contrast to the analysis of the ingroup dataset, statistical support for most of the basal nodes within the Limnochromini was low. The eight tested outgroup sets gave rise to five different tree topologies, with variability in root position, ingroup topology and bootstrap support (Fig. 2).

## 4 Discussion

### 4.1 Phylogenetic inference, rapid radiation and ecological diversification

Irrespective of the composition of the outgroup in the phylogenetic reconstruction, our AFLP data corroborated the mitochondrial evidence (Duftner et al., 2005) for a monophyletic clade consisting of the genera *Greenwoodochromis*, *Limnochromis*, *Baileychromis*, *Reganochromis*, *Triglachromis* and *Gnathochromis permaxillaris* [the nominally congeneric *G. pfefferi* belongs to the tribe Tropheini (Duftner et al., 2005), and a taxonomic revision is required]. A monotypic genus, *Tangachromis*, was not included in the genetic studies, but clustered within the Limnochromini according to morphological data (Takahashi, 2003). The derived monophyletic position of the genus *Greenwoodochromis* in both our nuclear and Duftner et al.’s (2005) mitochondrial phylogeny strongly supports its retention within Limnochromini and discards an earlier classification as Greenwoodochromini (Takahashi, 2003). The eponymous genus of the Limnochromini, *Limnochromis*, originally comprised multiple species, most of which have meanwhile been assigned to alternative genera (*Triglachromis*, *Gnathochromis*, *Tangachromis*, *Greenwoodochromis*; Poll, 1986; Takahashi, 2003). The taxonomy of the remaining three *Limnochromis* species remains problematic, since neither nuclear nor mitochondrial data support a monophyletic relationship, but separate *L. auritus* from *L. abeelei* and *L. staneri* in all phylogenetic reconstructions (Figs. 1 and 2).

The root of the Limnochromini tree could not be determined in this study. Different sets of outgroup taxa yielded different solutions, and midpoint rooting of the ingroup did not resolve the basal branching order. Both mtDNA and AFLP data suggest an almost simultaneous diversification of several ecologically distinct lineages at the onset of the Limnochromini radiation. Some well supported clades were resolved differently in the AFLP and mtDNA topologies. However, according to the SH test, these differences were not significant. Despite some well known AFLP-specific shortcomings, the genome-wide distribution of AFLP markers minimizes locus-specific effects potentially problematic for analyses of single or just a few loci (Koopman, 2005; Fink et al., 2010). In case of conflicting outcomes, it is therefore usually the multi-locus reconstruction that is considered a better approximation of the true evolutionary history than the mitochondrial gene tree (e.g., Kingston et al., 2009; Koblmüller et al., 2007a, 2010; Fink et al., 2010). According to the AFLP ingroup dataset, which is probably the most reliable representation of ingroup relationships given the obvious outgroup effect, one of the major lineages comprises *Baileychromis* and *Reganochromis*, benthic predators with elongated bodies and large eyes, which prey upon shrimp and small fish on deep muddy bottoms (Coulter, 1991). A second lineage consists of a single species, *G. permaxillaris*, a deepwater zooplankton feeder with a strongly enlarged upper lip and protractile mouth apparatus (Coulter, 1991; Konings, 1998). In the third lineage, *Greenwoodochromis christyi*, *G. bellcrossi*, *L. staneri* and *L. abeelei* are all true benthic deepwater fish. Both *Limnochromis* species are generalized predators on muddy flats, feeding on small fish, molluscs and arthropods (Coulter, 1991). The two *Greenwoodochromis* species occupy a slightly different habitat and feed on invertebrates and small fish in the transition between sandy and rocky bottoms (Konings, 1998). Finally, a somewhat less well supported clade comprises *L. auritus* and *T. otostigma*, whose habitat extends from muddy bottoms at the shores far into the depths of the lake (Coulter, 1991). *Triglachromis otostigma* is the only known detritus feeder among the Lake Tanganyika cichlids (Coulter, 1991), and uses the pectoral fin ray tips as sensory organs to screen the muddy ground, which is swallowed in large quantities when potential food items are detected.

The ecological segregation during the radiation of the Limnochromini is consistent with the proposition that in adaptive radiations, early bursts of cladogenesis in response to ecological opportunity are followed by a decline in the rate of speciation due to the filling of niches (Losos, 2010). Indeed, cladogenesis among and within Lake Tanganyika’s cichlid tribes appears to follow this pattern (Seehausen, 2006; Koblmüller et al., 2008a; Sturmbauer et al., 2011), and in particular in lineages unaffected by physical barriers to gene flow, ecological segregation may be an important promoter of diversification (Koblmüller et al., 2005; Kidd et al., 2006; Kirchberger et al., 2012).

### 4.2 Outgroup effect on root position, tree topology and bootstrap support

Outgroup choice is a crucial step in phylogenetic analysis, in particular when potential outgroup taxa are only distantly related to the ingroup. Regarding phylogenetic inference from DNA and protein sequence data, numerous studies showed that highly divergent outgroup taxa can underlie a loss of phylogenetic signal and can generate random outgroup effects as well as systematic biases by attracting fast evolving and compositionally similar ingroup species towards the base of the tree and thus heavily impact tree topology and root position (Wheeler, 1990; Foster and Hickey, 1999; Huelsenbeck et al., 2002; Shavit et al., 2007; Rota-Stabelli and Telford, 2008; Rosenfeld et al., 2012). Surprisingly, potential effects of outgroup choice on phyogenetic inference have been largely ignored in AFLP-based studies. However, homoplastic non-homologous bands in outgroup and ingroup taxa might lead to the same effects as homoplasy in DNA and protein sequences. Indeed, analyses of simulated AFLP data provided evidence for a dramatic decrease of phylogenetic accuracy, both with respect to tree topology and relative branch length, with increasing divergence within datasets, which was due to a rapid accumulation of non-homologous AFLP bands (e.g., Althoff et al., 2007; García-Pereira et al., 2010). Likewise, examination of empirical data shed doubt on the usefulness of AFLPs for the resolution of deep phylogenetic relationships (Near and Keck, 2012).

The impact of outgroup composition on root position, bootstrap support and tree topology in our AFLP dataset likely originates from the combination of the large genetic distance between the available outgroup taxa and the ingroup on the one hand, and the short basal branches within the ingroup on the other hand. Moreover, considerable differences in branch lengths between ingroup and some outgroup taxa might promote random outgroup effects (Wheeler, 1990; Huelsenbeck et al., 2002). With an age of 5–6 MY (or more, contingent on the calibration; Genner et al., 2007; Schwarzer et al., 2009; but see Koblmüller et al., 2008a,b), the split between the Limnochromini and the outgroup taxa is approximately twice as old as the most recent common ancestor of the extant limnochromines, from which the major limnochromine lineages radiated almost simultaneously (Duftner et al., 2005; present data). This allowed different sets of outgroup taxa to attach to different branches of the ingroup and pull certain ingroup taxa towards the base of the tree, which naturally affected bootstrap support and topology of the ingroup clades.

Prompted by these observations, we examined whether previous AFLP-based phylogenetic reconstructions of various Lake Tanganyika cichlid lineages were robust to the removal of the outgroup. No substantial outgroup effect on inferences of ingroup relationships was detected in these datasets (Koblmüller et al., 2007b, 2010; Kirchberger et al., 2012), as changes in branching order concerned only those clades, which had low statistical support irrespective of whether or not the outgroup was included in the analyses (data not shown). Notably, in none of these datasets was the basal ingroup radiation as rapid and the relative divergence from the outgroup as large as it was in the Limnochromini dataset. However, outgroup effects may underlie the different AFLP tree topologies of the Perissodini radiation obtained by Koblmüller et al. (2007b) and Takahashi et al. (2007), as the latter study used some highly divergent outgroup taxa.

## 5 Conclusions

The effect of outgroup composition on our analyses prompts us to call for increased awareness of potential outgroup problems in work with empirical data, and to suggest that additional theoretical work on the causes and consequences of outgroup effects is warranted, specifically with regard to AFLP data. Outgroup choice is often confounded by the lack of sufficiently closely related taxa and the present study also demonstrates that particular care has to be taken in such instances. With regard to implications for the taxonomy of Limnochromini, AFLP data are in accordance with previous mtDNA sequence data (Duftner et al., 2005) in that the genus *Greenwoodochromis* is nested in the Limnochromini rather than constituting a separate tribe, as well as regarding the polyphyly of the genus *Limnochromis*. Finally, ecological segregation appears to have occurred at the early stage of the limnochromine radiation.

## Figures and Tables

**Fig. 1:**
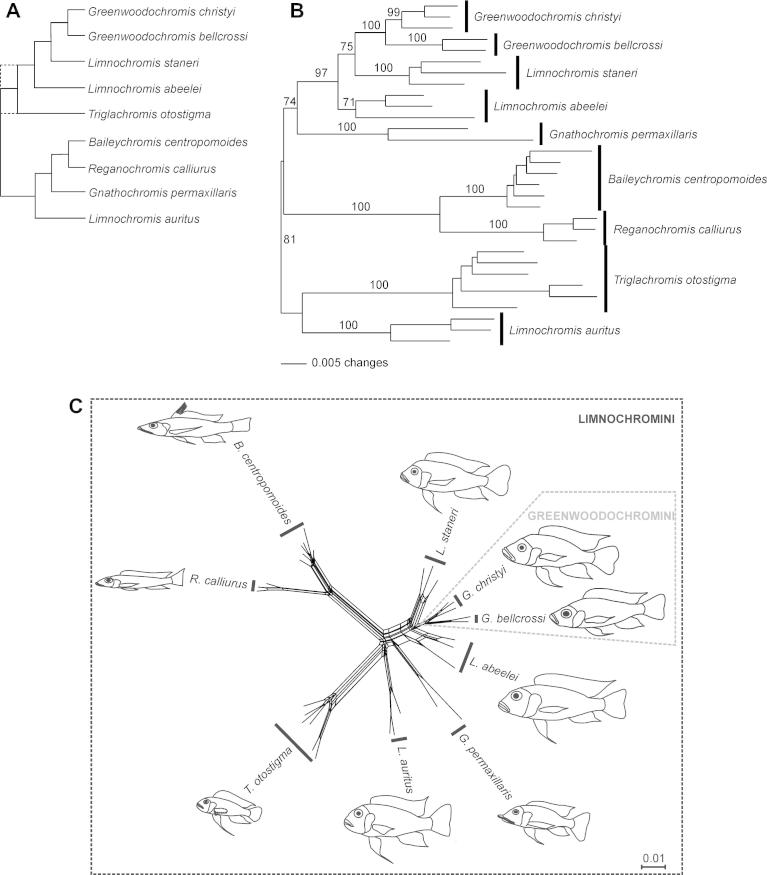
Phylogenetic relationships of the Limnochromini. (A) Mitochondrial phylogeny of the Limnochromini according to Duftner et al. (2005). Solid lines represent the topology obtained by maximum likelihood, dotted lines show the strict consensus of neighbor-joining, maximum likelihood and maximum parsimony trees. (B) Nuclear (AFLP) phylogeny of the Limnochromini; NJ tree based on Nei & Li distances (Nei and Li, 1979) with bootstrap values shown at the branches. (C) Neighbor-Net graph based on Nei & Li distances.

**Fig. 2:**
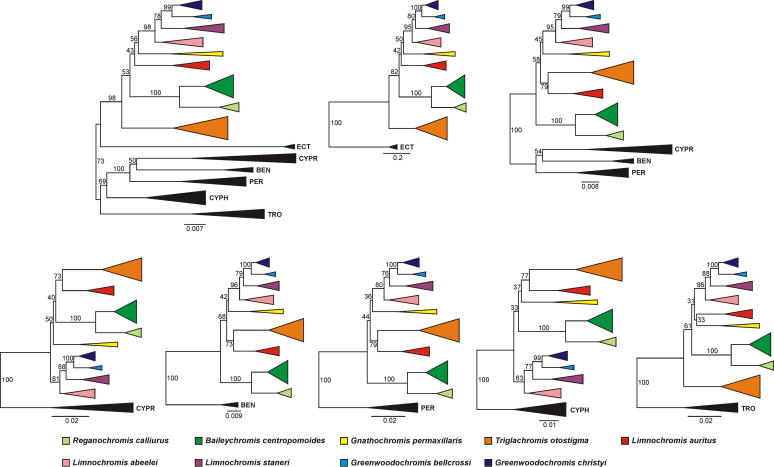
Random outgroup effect on root position, tree topology and bootstrap support. NJ trees based on Nei & Li distances (Nei and Li, 1979) using different sets of outgroup taxa (TRO, Tropheini; CYPH, Cyphotilapiini; PER, Perissodini; BEN, Benthochromini; CYPR, Cyprichromini; ECT, Ectodini), with bootstrap values shown at the branches.

## Appendix A Supplementary material

Supplementary data 1 Supplementary material contains Supplementary Table 1.
