# Supplementary material for: Outgroup effects on root position and tree topology in the AFLP phylogeny of a rapidly radiating lineage of cichlid fish
Source: Mol Phylogenet Evol. 2014 Jan;70:57–62. doi: 10.1016/j.ympev.2013.09.005 (PMC3842234; doi:10.1016/j.ympev.2013.09.005)
Supplement: Supplementary file 1 [file mmc1.pdf]

Supplementary Table 1

Sample list.

Species	SampleID	Origin	Photo	Voucher
**Ingroup**				
Greenwoodochromis bellcrossi	13429	Aquarium trade	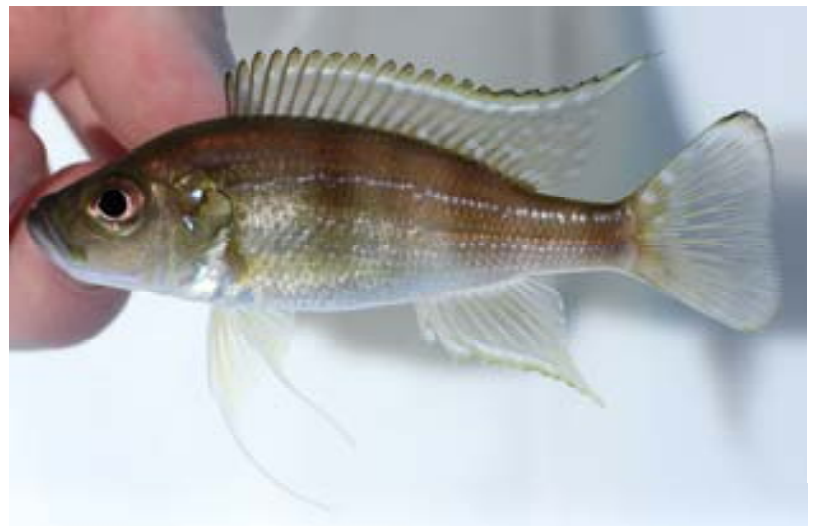	
	13430	Aquarium trade		
Greenwoodochromis christyi	13367	Mpulungu fishmarket	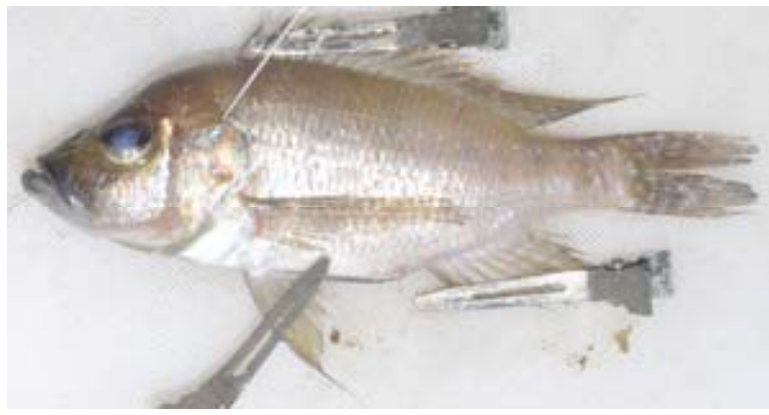	
	13390	Mpulungu fishmarket		
	13399	Mpulungu fishmarket	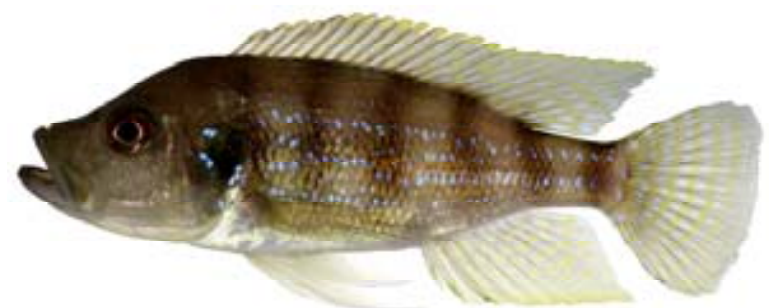	voucher in Graz (T03 3/B8)
Limnochromis staneri	12951	Mpulungu fishmarket	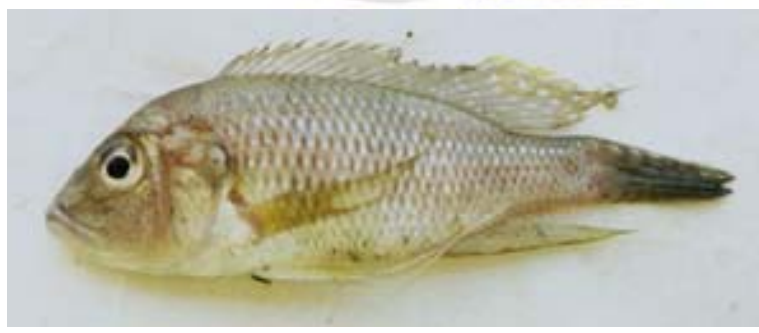	voucher in Graz (T03 5/J10)

	13365	Mpulungu fishmarket	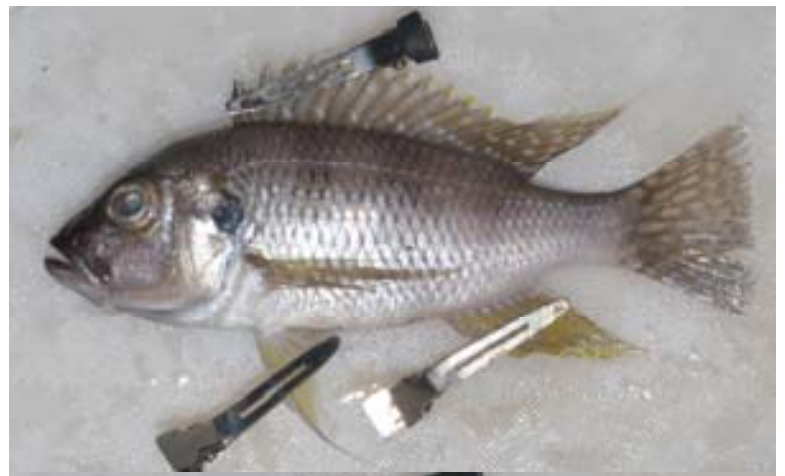	
	13366	Mpulungu fishmarket	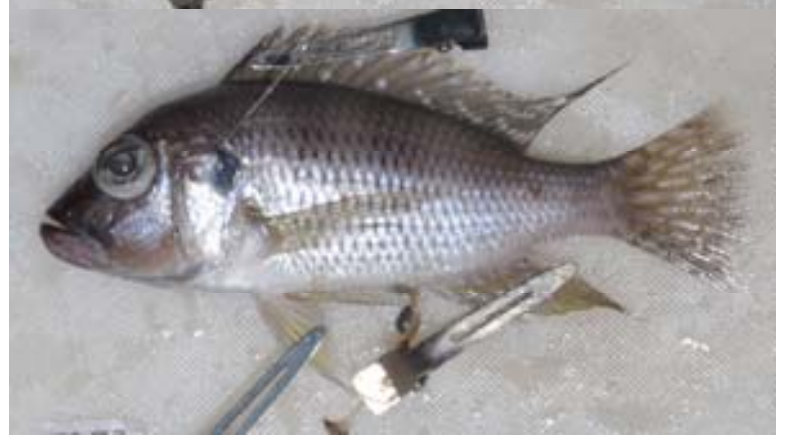	
Limnochromis abeelei	12943	Mpulungu fishmarket		
	13369	Mpulungu fishmarket	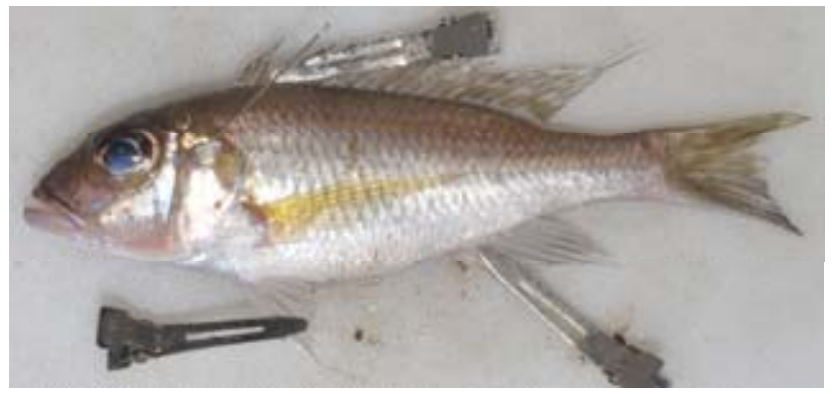	
	13483	Mpulungu fishmarket		
Gnathochromis permaxillaris	13364	Mpulungu fishmarket	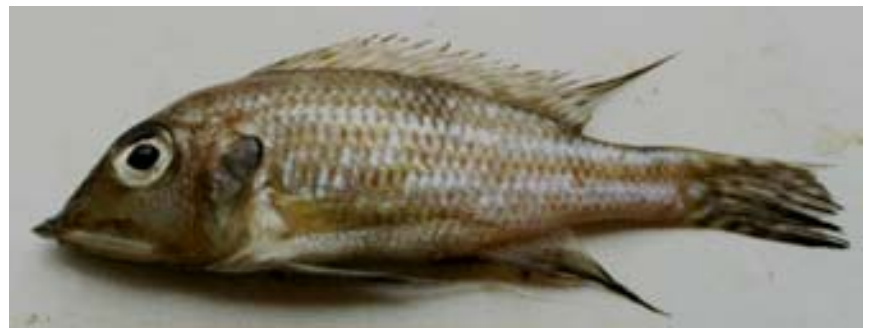	
	13467	Aquarium trade		voucher in Graz (T01 7/H9)
Limnochromis auritus	12953	Mbita (west)	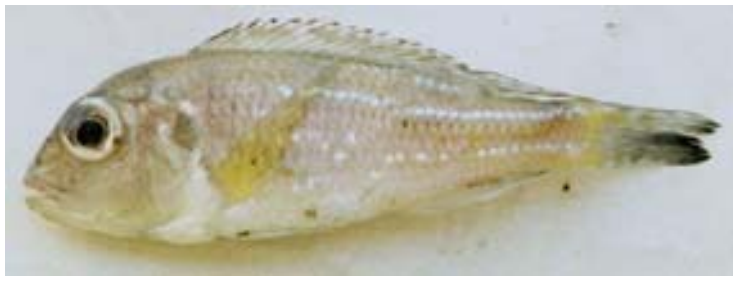	voucher in Mpulungu (T03 6/A3)

	13373	Mpulungu fishmarket	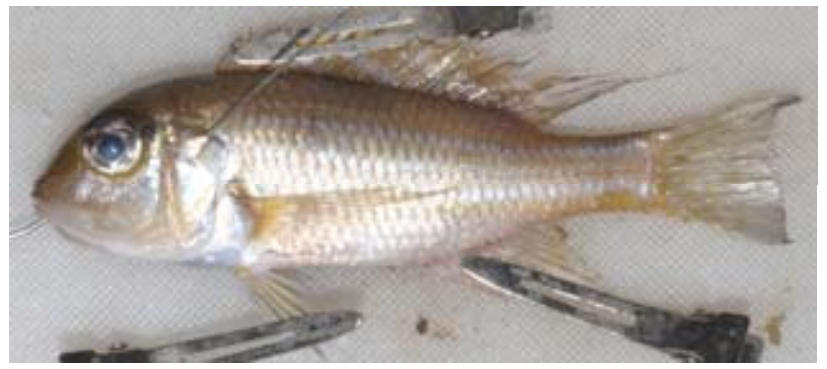	
	13473	Chituta Bay		
Baileychromis centropomoides	12961	Mpulungu fishmarket	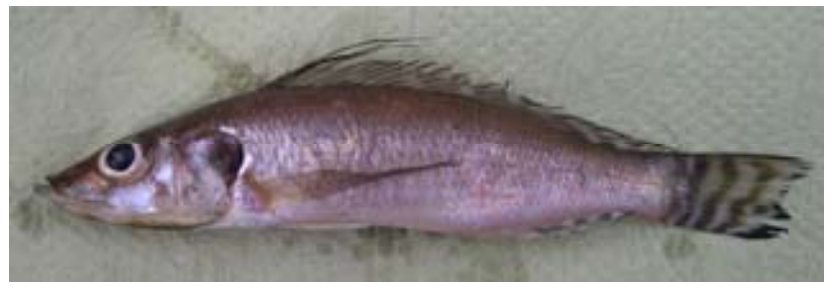	
	12962	Mpulungu fishmarket		
	13417	Mpulungu fishmarket	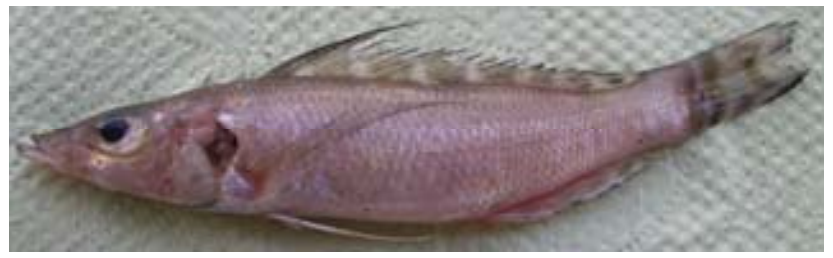	
	13418	Mpulungu fishmarket		
	13419	Mpulungu fishmarket		
	13420	Mpulungu fishmarket		voucher in Graz (T06 10/A2)
Reganochromis calliurus	13362	Aquarium trade	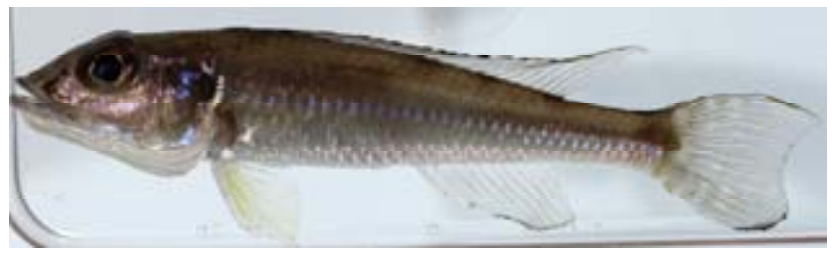	
	13484	Aquarium trade		voucher in Graz (T10 10/A6)
	13485	Aquarium trade		
Triglachromis otostigma	13359	Aquarium trade		voucher in Graz (T09 20/H6)
	13360	Aquarium trade		voucher in Graz (T09 20/H7)
	13371	Mpulungu fishmarket	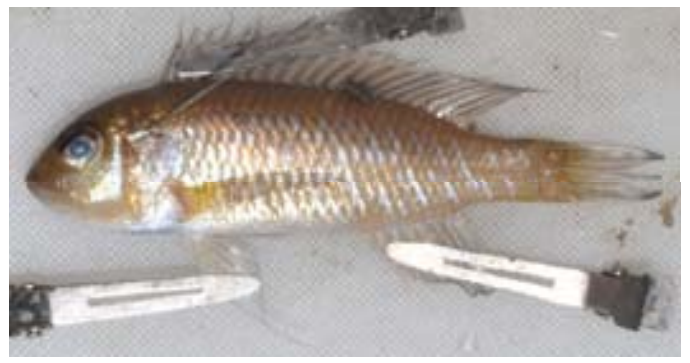	

	13372	Mpulungu fishmarket	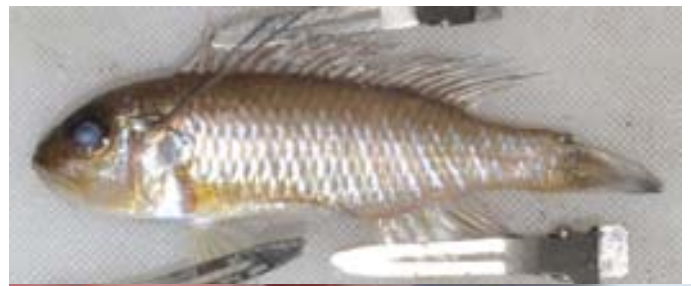	
	13434	Aquarium trade	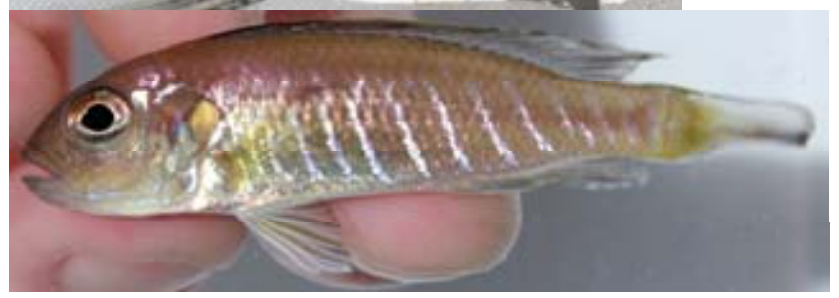	voucher in Graz (KS4 H6)
	13435	Aquarium trade		
**Outgroup**				
Xenotilapia ochrogenys	13052	Aquarium trade		
	13051	Aquarium trade		
Cyprichromis leptosoma	12981	Kasenga Rocks		
Cyprichromis microlepidotus	12988	Aquarium trade		
Paracyprichromis nigripinnis	13091	Aquarium trade		
Benthochromis tricoti	13358	Kalambo Lodge		
Benthochromis melanoides	13357	Sumbu (north)		
Perissodus microlepis	13356	Kalambo Lodge		
	T0631F5	Mbita (east)		
Haplotaxodon microlepis	13438	Lufubu estuary		
Ctenochromis benthicola	13437	Aquarium trade		
	13436	Aquarium trade		
Cyphotilapia frontosa	13439	Lufubu estuary		
	13440	Lufubu estuary		
Tropheu smoorii	KS4F10	Murago		
	KS4C1	Murago		
Tropheus duboisi	13361	Aquarium trade		

Coordinates: Chituta Bay, 8°45’ S, 31°10’ E; Kalambo Lodge, 8°37' S, 31°37' E; Kasenga Rocks, 8°44' S, 31°09' E; Lufubu estuary, 8°32' S, 30°44' E; Mbita (east), 8°45’ S, 31°06’ E; Mbita (west), 8°45’ S, 31°05’ E; Murago, 7°05’ S, 29°50’ E; Sumbu (north), 8°31’ S, 30°28’ E.

Note that fish obtained at the fishmarket in Mpulungu might have been caught anywhere in southern Lake Tanganyika.
